# Clinical characteristics of hypertrophic cranial pachymeningitis in granulomatosis with polyangiitis: a retrospective single-center study in China

**DOI:** 10.1186/s13075-023-03239-9

**Published:** 2024-01-02

**Authors:** Yiran Chen, Lijuan Gou, Li Wang, Min Qian, Qingjun Wu, Wenjie Zheng, Mengtao Li, Xiaofeng Zeng, Fengchun Zhang

**Affiliations:** 1 Department of Rheumatology and Clinical Immunology, Peking Union Medical College Hospital, Chinese Academy of Medical Sciences & Peking Union Medical College, Key Laboratory of Rheumatology and Clinical Immunology, Ministry of Education, National Clinical Research Center for Dermatologic and Immunologic Diseases, Beijing, China; 2 Department of Pediatrics Rheumatology and Clinical Immunology, Peking Union Medical College Hospital, Chinese Academy of Medical Sciences, Beijing, China; 3 Department of Neurology, Peking Union Medical College Hospital, Chinese Academy of Medical Sciences, Beijing, China

**Keywords:** Granulomatosis with polyangiitis, Hypertrophic cranial pachymeningitis, Meningitis, Neurologic manifestations, Antibodies, Antineutrophil cytoplasmic

## Abstract

**Background:**

Hypertrophic cranial pachymeningitis (HCP) is uncommon but a poorly understood complication of granulomatosis with polyangiitis (GPA).

**Objectives:**

We conducted this retrospective study to elucidate the clinical characteristics and factors independently associated with granulomatosis with polyangiitis (GPA) complicated by hypertrophic cranial pachymeningitis (HCP) in China.

**Methods:**

We collected the medical records of 78 patients diagnosed with GPA who were admitted to the inpatient department of Peking Union Medical College Hospital between January 2003 and September 2021. Clinical features, laboratory and radiological findings, and Birmingham Vasculitis Activity Scores (excluding meningitis score) were recorded. A binary logistic regression analysis was performed to analyze factors independently associated with GPA-related HCP.

**Results:**

Headache (100%) and cranial nerve palsy (61.5%) were common manifestations of HCP. Compared to 52 GPA patients without HCP, 26 patients with HCP required more time from initial symptoms to diagnosis, with a lower ratio of pulmonary and renal involvement, a higher ratio of myeloperoxidase–antineutrophil cytoplasmic antibody (MPO-ANCA) positivity, conductive or sensorineural hearing loss, mastoiditis, and decreased vision or sudden visual loss. Binary logistic regression analysis indicated that proteinase 3–antineutrophil cytoplasmic antibody (PR3-ANCA) negativity (OR 10.698, *p* = 0.001), conductive or sensorineural hearing loss (OR 10.855, *p* = 0.005), and decreased vision or sudden visual loss (OR 8.647, *p* = 0.015) were significantly associated with GPA-related HCP. Of the 26 patients, 18 received methylprednisolone pulse treatment, and 18 received intrathecal injections of dexamethasone and methotrexate.

**Conclusions:**

HCP was a severe manifestation of GPA in our study. Independent factors associated with the occurrence of HCP in patients with GPA included PR3-ANCA negativity, conductive or sensorineural hearing loss, and decreased vision or sudden visual loss. Furthermore, GPA-related HCP was associated with higher disease activity, requiring more intensive treatments.

**Supplementary Information:**

The online version contains supplementary material available at 10.1186/s13075-023-03239-9.

## Introduction

Granulomatosis with polyangiitis (GPA), one of three types of antineutrophil cytoplasmic antibody (ANCA)-associated vasculitides, is an autoimmune disease characterized by inflammation in small vessels, which primarily affects the ear, nose, larynx, lung, kidney, heart, gastrointestinal tract, skin, peripheral nervous system, and central nervous system (CNS). CNS involvement is rare (affecting 11.7% of patients) but significant in GPA, and it was thus included in the 1996 five-factor score (FFS) [1]. Hypertrophic pachymeningitis (HP) comprises two forms: hypertrophic cranial pachymeningitis (HCP) and hypertrophic spinal pachymeningitis (HSP). HCP is the most prevalent CNS manifestation, occurring in 36–46% of patients with GPA with CNS involvement [1, 2].

HCP, depicted as localized or diffuse thickening and enhancement of the dura mater on contrast-enhanced magnetic resonance images (MRIs), with histopathology of granulomatous inflammation and necrotizing vasculitis, has been increasingly reported in patients with ANCA-associated vasculitis and occurs most frequently in patients with GPA [3]. Patients usually present with headache as the initial symptom, with or without cranial nerve deficits, which increases the difficulty of diagnosing GPA-related HCP. A detailed analysis of the Chinese GPA population with HCP has not been performed. Therefore, we analyzed 26 patients with confirmed GPA-related HCP in a single center in China to investigate the clinical features and treatment of GPA with HCP and to explore independent associated factors to facilitate early diagnosis.

## Methods

### Patients

**Fig. 1:**
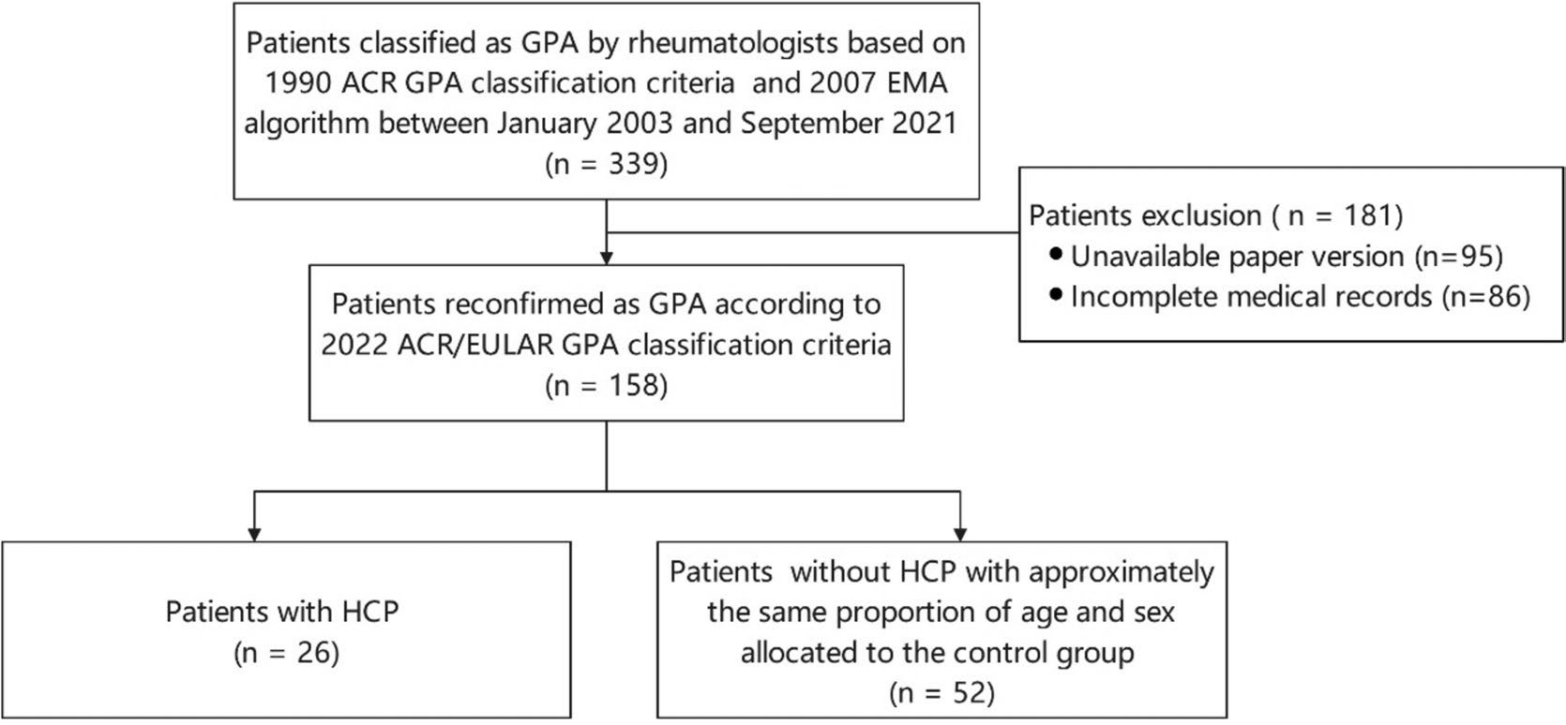
Patient flow diagram

In this retrospective study conducted at the inpatient department of Peking Union Medical College Hospital (PUMCH) between January 2003 and September 2021, a total of 339 patients diagnosed with granulomatosis with polyangiitis (GPA) were initially classified using both the American College of Rheumatology (ACR) 1990 classification criteria [4] and the 2007 European Medicines Agency (EMA) algorithm [5]. Because it is difficult to investigate the cumulative manifestations and laboratory assessment of these patients, we searched the electronic medical record system and recorded data at the time-point of initial diagnosis. During the study period, 30.7% of GPA patients had eye involvement, 91.4% had ENT involvement, 83.2% had pulmonary involvement, and 34.8% had renal involvement. At this initial diagnosis, PR3-ANCA was the predominant type in GPA, accounting for 73.4% of cases. Because the period covered is as long as 18 years, 158 patients with complete medical records were reconfirmed as GPA based on the 2022 ACR/European League Against Rheumatism (EULAR) GPA classification criteria by two rheumatologists [6] (Fig. 1).

Subsequently, we analyzed 26 patients with GPA and HCP from the population, and 52 patients diagnosed with GPA without HCP with approximately the same proportion of age and sex were allocated to the control group using a random number table.

### Clinical and laboratory assessment

We systematically evaluated HCP according to clinical manifestations, laboratory tests of cerebrospinal fluid (CSF), and physical examinations, including contrast-enhanced MRIs of the brain and spinal cord. There has been no change in the standard values and measurement methods for laboratory assessment from 2003 to 2021. The Birmingham Vasculitis Activity Score (BVAS) was utilized to assess the disease activity before the initiation of induction therapy. Two authors independently evaluated patients’ BVAS according to BVAS version 3.0. It is noted that meningitis scores were not calculated. Each patient was scored for prognosis assessment according to the 2009 revised FFS system. Median disease duration was defined as the interval from the onset of initial symptoms to GPA-related HCP diagnosis.

Laboratory tests included erythrocyte sedimentation rate (ESR), hypersensitive C-reactive protein (hsCRP), white blood cell (WBC) count, and platelet count. Perinuclear and cytoplasmic patterns were differentiated using immunofluorescence assay (IFA). Myeloperoxidase (MPO) and proteinase 3 (PR3)-ANCA levels were assessed using the chemiluminescent immunoassay (CLIA). Abnormal urinary sediment was defined as red cell casts or more than five red blood cells with an abnormal form per high-power field. Twenty-six patients underwent lumbar puncture, and CSF pressure, protein levels, and WBC counts were recorded. Considering the invasive lesions and potential risks of biopsy, only one patient was diagnosed with HCP by biopsy in our study; other patients were diagnosed through contrast-enhanced MRI. Diffusion-weighted, T2-weighted, fluid-attenuated inversion recovery (FLAIR), T1-weighted, and gadolinium-enhanced images were obtained using contrast-enhanced MRI in patients with GPA-related HCP. Diffuse pachymeningitis was defined as the involvement of more than two non-contiguous sites, whereas focal pachymeningitis indicated the involvement of only one site.

### Treatment strategy

At the time of diagnosis, patients received proper therapy, and the medications used throughout the induction period as well as treatment responses were recorded in detail. Methylprednisolone pulse was administered at 0.5 ~ 1.0 g/day for 3 ~ 5 days. Intravenous immunoglobulin (IVIG) was given at 20 g/day for 3 ~ 5 days. And intrathecal injections of dexamethasone (10 mg each time) and methotrexate (10 mg each time) were also administered. The response to treatment was assessed by symptoms, laboratory tests, and imaging with contrast-enhanced MRIs to identify changes in the thickening of the dura mater. Outcomes of interest were remission within 6 months, relapse during observation time, and death within 1 year. Remission was defined as the absence of typical signs, symptoms, or other features of active AAV. GPA relapse was defined as clinical signs of vascular activity in any organ system after complete disease remission through standard induction therapy [7].

### Statistical analysis

Statistical analyses were performed using SPSS version 25.0. Kolmogorov–Smirnov tests were used to assess for normal distribution. Continuous normally distributed data are expressed as the mean ± standard deviation (SD), and non-normally distributed data are expressed as the median and interquartile range (IQR). Univariate analysis included chi-square or Fisher’s exact tests for categorical variables and *t*-tests and Mann–Whitney tests for continuous variables to identify associated variables. Significant variables from the univariate analysis underwent binary logistic regression analysis using the forward conditional method to identify independent factors.

In the model, PR3-ANCA negativity, conductive or sensorineural hearing loss, and decreased or sudden visual loss were identified as statistically significant variables, consistent with previous studies indicating that conductive hearing loss and sudden visual loss were significant factors in patients with HP related to AAV [8]. Therefore, these three covariates were selected to ensure statistical robustness, especially given the small sample size, while balancing clinical and statistical considerations. *P* values for other excluded variables, as well as an alternative model that includes all the significant variables from the univariate analysis using the “enter” method, were presented in Supplementary Tables 1 and 2. To assess the predictive capacity of the model, the receiver operating characteristic curve (ROC) was plotted and the area under the curve (AUC) was reported.

## Results

### Features of HCP in GPA

**Fig. 2:**
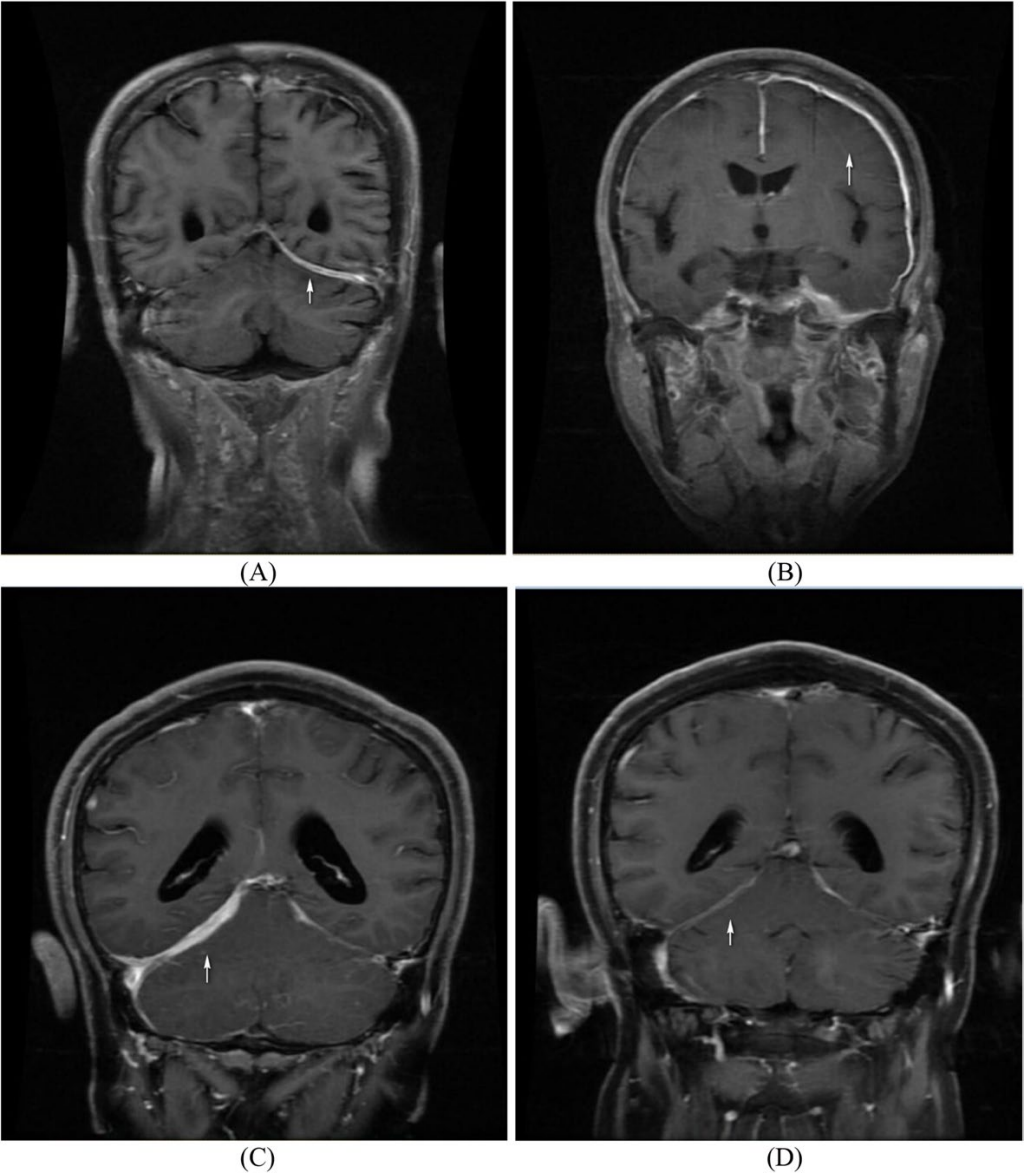
Brain magnetic resonance imaging of patients with GPA and HCP. The dural thickness of the left tentorium cerebella and left frontotemporal lobe are indicated by the arrows in (**A**) and (**B**) on T1-weighted gadolinium-enhanced images. The changes of tentorium cerebelli before and after treatment in the same patient are shown in (**C**) and (**D**). GPA, granulomatosis with polyangiitis; HCP, hypertrophic cranial pachymeningitis

The neurological manifestations and neuroimaging findings of patients with HCP are shown in Table 1 and Fig. 2. HCP primarily presented with severe headache, which was reported in 100% of patients in the HCP group. The second most common neurological manifestation was cranial nerve palsy (61.5%). In patients with cranial nerve palsy, the most common findings were involvement of the optic and facial nerves (each *n* = 7, 26.9%), followed by trigeminal nerve palsy (*n* = 6, 23.1%) and vestibulocochlear nerve palsy (*n* = 5, 19.2%). Four patients had pituitary involvement, and two of them presented with polydipsia and polyuria. As for neuroimaging, 22 patients (84.6%) had diffuse hypertrophic pachymeningitis, and four patients (15.4%) showed focal dural thickening with gadolinium enhancement. The convexity (46.4%) was the most frequently involved site, followed by the skull base (*n* = 11, 42.3%), tentorium (*n* = 9, 34.6%), falx (*n* = 5, 19.2%), and sellar region (*n* = 2, 7.7%). In total, 23 patients with GPA-related HCP underwent lumbar puncture, and five of them exhibited increased intracranial pressure, ranging from 220 to > 330 mmH_2_O. Among these five patients, four had a small increase in CSF protein levels. The median CSF WBC count was 2 cells/L.

**Table 1:** Neurologic manifestations and neuroimaging findings in patients with GPA-related HCP

Number of patients	26
Neurologic involvement [*n* (%)]	
CNS involvement	26 (100.0%)
PNS involvement	4 (15.4%)
Neurologic involvement as initial manifestation	4 (15.4%)
Neurologic manifestations of CNS involvement [*n* (%)]	
Headache	26 (100.0%)
Cranial nerve palsy	16 (61.5%)
Multiple CN involvement	
CN I	0 (0%)
CN II	7 (26.9%)
CN III	3 (11.5%)
CN IV	2 (7.7%)
CN V	6 (23.1%)
CN VI	3 (11.5%)
CN VII	7 (26.9%)
CN VIII	5 (19.2%)
CN IX	2 (7.7%)
CN X	2 (7.7%)
CN XI	0 (0%)
CN XII	2 (7.7%)
Pituitary involvement	4 (15.4%)
Neuroimaging findings indicating HCP	26 (100.0%)
Diffuse HCP	22 (84.6%)
Sites of HCP involvement [*n* (%)]	
Falx	5 (19.2%)
Convexity	12 (46.2%)
Tentorium	9 (34.6%)
Skull base	11 (42.3%)
Sellar region	2 (7.7%)
CSF pressure^a^[mmH2O], median (IQR)	155 (125, 185)
CSF protein^b^[mg/dL], median (IQR)	49 (34.75, 65.25)
CSF WBC count^b^[leukocytes/mm^3^], median (IQR)	2 (0, 6.75)

*GPA* granulomatosis with polyangiit is, *HCP* hypertrophic cranial pachymeningitis, *CNS* central nervous system, *PNS *peripheral nervous system, *CN* central nerve, *CSF* cerebrospinal fluid, *WBC* white blood cells, *IQR* interquartile range

^a^CSF pressure data available for 23 patients

^b^CSF protein and CSF WBC count data available for 22 patients

### Baseline characteristics of GPA patients with or without HCP

We compared baseline clinical characteristics of patients with and without HCP (Table 2). The median age of patients with GPA-related HCP was 51 years. The time between the onset of symptoms and GPA diagnosis was longer in those with HCP than in those without (12 (3, 23.25) versus 3 (1, 7.75) months, *p* = 0.001). The median disease duration in patients with GPA-related HCP was 16 months, ranging from 3 to 191 months. Weight loss, fever, arthritis or joint pain, and myalgia were observed in 53.8%, 42.3%, 0.0%, and 7.7% of patients with GPA-related HCP, respectively. Patients with HCP had visual impairment (34.6%), ear, nose, and throat (ENT) involvement (92.3%), pulmonary involvement (61.5%), and renal involvement (11.5%). Notably, conductive or sensorineural hearing loss (83.5%), sinusitis (73.1%), and mastoiditis (57.7%) were prominent and common symptoms in these patients. The first onset of presentations included neurological symptoms in 15.4% (*n* = 4), visual symptoms in 7.7% (*n* = 2), and pulmonary involvement in 3.8% (*n* = 1). Four patients (15.4%) had peripheral neuropathy during GPA-related HCP, which was similar to patients without HCP. Twenty-two patients with GPA and HCP tested positive for ANCA, including 12 cases who were PR3-ANCA positive and 7 cases who were MPO-ANCA positive. Patients with HCP, when compared with those without HCP, had a higher proportion of decreased vision or sudden visual loss (34.6% versus 3.8%, [*p* = 0.001]) and more frequent conductive or sensorineural hearing loss and mastoiditis (83.5% versus 50.0%, 57.7% versus 28.8%, [*p* = 0.001, *p* = 0.014]). Patients also had less frequent pulmonary and renal involvement (61.5% versus 86.5%, 11.5% versus 36.5%, [*p* = 0.012, *p* = 0.021]) and less frequent abnormal urinary sediment and elevated serum creatinine (11.5% versus 34.6%, 0.0% versus 23.1%, [*p* = 0.030, *p* = 0.020]). In addition, individuals tested more MPO-ANCA-positive (26.9% versus 3.8%, *p* = 0.009) and less PR3-ANCA-positive (46.2% versus 84.6%, *p* < 0.001). However, the ratio of the FFS ≥ 1 in the two groups was similar. Excluding the meningitis score, BVAS in the HCP group was found to be slightly higher than in the non-HCP group. However, the difference in BVAS between the two groups did not reach statistical significance.

**Table 2:** Comparison of baseline features between GPA patients with HCP and without HCP

Characteristics	GPA with HCP *n* = 26	GPA without HCP *n* = 52	*p-*values
Demographics			
Age, median (IQR)	51 (37.5, 60.25)	43 (27.25, 55.75)	0.084
Gender (male/female, *n*)	29/23	14/12	0.872
Time from initial symptoms to GPA diagnosis (month), median (IQR)	12 (3, 23.25)	3 (1, 7.75)	0.001^*^
Clinical manifestation [*n* (%)]			
Fever	11 (42.3)	23 (44.2)	0.872
Weight loss	14 (53.8)	21 (40.4)	0.260
Myalgia	2 (7.7)	3 (5.8)	1.000
Mucous membrane/eyes involvement	12 (46.2)	20 (38.5)	0.515
Orbital pseudotumor	5 (19.2)	6 (11.5)	0.565
Decreased vision/sudden visual loss	9 (34.6)	2 (3.8)	0.001^*^
Conjunctivitis/uveitis/blepharitis/keratitis	1 (3.8)	12 (23.1)	0.05
ENT involvement	24 (92.3)	48 (92.3)	1.000
Sinusitis	19 (73.1)	47 (90.4)	0.096
Conductive or sensorineural hearing loss	23 (83.5)	26 (50.0)	0.001^*^
Mastoiditis	15 (57.7)	15 (28.8)	0.014^*^
Subglottic stenosis	1 (3.8)	1 (1.9)	1.000
Pulmonary involvement	16 (61.5)	45 (86.5)	0.012^*^
Renal involvement	3 (11.5)	19 (36.5)	0.021^*^
Abnormal urinary sediment	3 (11.5)	18 (34.6)	0.030^*^
Elevated serum creatinine	0 (0.0)	12 (23.1)	0.020^*^
Peripheral neuropathy	4 (15.4)	7 (13.5)	1.000
Clinical score BVAS (without meningitis scores) [mean, SD]	17.31 ± 4.116	14.98 ± 6.229	0.053
FFS ≥ 1[*n* (%)]	4 (15.4)	16 (30.8)	0.142
Laboratory findings			
ANCA positivity [*n* (%)]	22 (84.6)	50 (96.2)	0.091
MPO-ANCA [*n* (%)]	7 (26.9)	2 (3.8)	0.009^*^
PR3-ANCA [*n* (%)]	12 (46.2)	44 (84.6)	< 0.001^*^
ESR, median (IQR) [mm/h]	64.00 (21.00, 98.25)	51.50 (17.25, 91.00)	0.433
hsCRP, median (IQR) [mg/L]	32.67 (5.86, 103.00)	23.19 (4.62, 79.13)	0.518

*GPA* granulomatosis with polyangiitis, *HCP* hypertrophic cranial pachymeningitis, *ENT involvement* ear, nose, and throat involvement, *BVAS* Birmingham Vasculitis Activity Score, *FFS* five-factor score, *ANCA* antineutrophil cytoplasmic antibody, *MPO* myeloperoxidase, *PR3* protease 3, *ESR* erythrocyte sedimentation rate, *hsCRP* hypersensitive C-reactive protein, *x* ± *S* mean ± standard deviation, *IQR* interquartile range

^*^p < 0.05

### Independently associated factors for GPA-related HCP patients

**Fig. 3:**
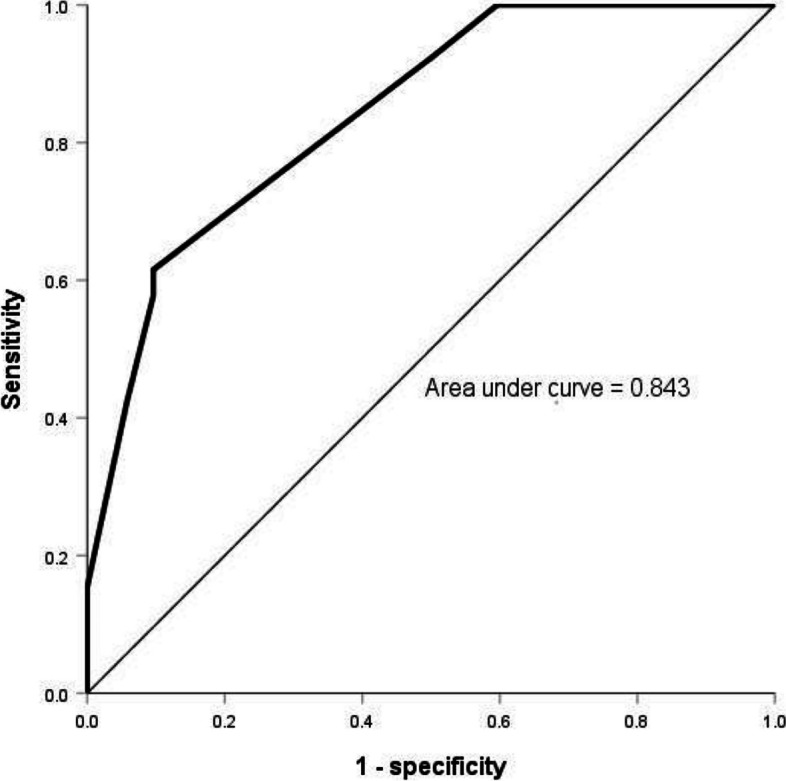
Receiver operator characteristic (ROC) curve analysis for GPA and HCP

We identified independent factors associated with GPA-related HCP using binary logistic regression analysis (Table 3), which included PR3-ANCA negativity (odds ratio [OR] 10.698, 95% confidence interval [CI] 2.547–44.923, *p* = 0.001), conductive or sensorineural hearing loss (OR 10.855, 95% CI 2.018–58.389, *p* = 0.005), and decreased vision or sudden visual loss (OR 8.647, 95% CI 1.519–49.241, *p* = 0.015). The optimal cutoff was 0.519 from the logistic prediction model to distinguish patients with GPA-related HCP from those without. The sensitivity of the model was 61.5% and the specificity was 90.4%. The area under curve (AUC) of HCP was 0.843 (CI 0.755–0.931) (Fig. 3).

**Table 3:** Binary logistic regression analysis for GPA patients with HCP

	OR (95% CI)	*p*-values
PR3-ANCA negativity	10.698 (2.547, 44.923)	0.001^*^
Conductive/sensorineural hearing loss	10.855 (2.018, 58.389)	0.005^*^
Decreased vision/sudden visual loss	8.647 (1.519, 49.241)	0.015^*^

*GPA* granulomatosis with polyangiitis, *HCP* hypertrophic cranial pachymeningitis, *PR3-ANCA* proteinase 3–antineutrophil cytoplasmic antibody, *OR* odds ratio, *CI* confidence interval

^*^p < 0.05

### Treatment

All patients with HCP (*n* = 26, 100%) were treated with glucocorticoids and cyclophosphamide (CYC) during the induction phase, and headaches were alleviated after 2 weeks to 1 month of treatment. The comparison of treatment strategies during the induction phase between patients with GPA with and without HCP is summarized in (Table 4). Methylprednisolone pulses were used more frequently in patients with GPA-related HCP (69.2% versus 34.6%, *p* = 0.004). Moreover, 69.2% of patients received intrathecal injections of dexamethasone and methotrexate for more severe CNS involvement. During the observation period, 27 patients without HCP received rituximab (RTX). Sixteen patients switched to RTX due to relapse, eight due to side effects of CYC, including infection, bladder bleeding, elevated transaminase levels, and profound gonadal toxicity, and three due to failure to achieve remission. The use of RTX was comparable in the groups with and without HCP (30.8% versus 51.9%, *p* = 0.077). Three patients in the HCP group switched to RTX due to CYC failure; three were due to side effects of cyclophosphamide; two were due to relapse. In addition, no statistically significant difference in the utilization of IVIG was observed between patients with HCP and without HCP (30.8% versus 11.5%, *p* = 0.077). 88.5% of patients with HCP and 92.3% of patients without HCP achieved remission within 6 months, with no significant difference in remission rates between the groups. Three patients with HCP and three patients without HCP achieved remission subsequently after switching to RTX. The median observation time for relapse was 24.79 months (ranging from 6 to 108 months). However, 61.5% of patients with HCP relapsed, compared to 44.2% of patients without HCP. Only one patient in the GPA without HCP group, who refused treatment with CYC or RTX during the induction phase, died of diffuse alveolar hemorrhage during 1 year of observation. No significant differences in recurrence or death were found between the groups.

**Table 4:** Treatment and outcomes of GPA patients with and without HCP

	GPA with HCP *n* = 26	GPA without HCP *n* = 52	*p-*values
Treatment, [*n* (%)]			
MP pulse	18 (69.2)	18 (34.6)	0.004^*^
CYC	26 (100)	49 (94.2)	0.532
Rituximab	8 (30.8)	27 (51.9)	0.077
IVIG	8 (30.8)	6 (11.5)	0.076
Intrathecal injection of methotrexate and dexamethasone	18 (69.2)	0 (0.0)	< 0.001^*^
Outcome, [*n* (%)]			
Remission	23 (88.5)	48 (92.3)	0.889
Recurrence	16 (61.5)	23 (44.2)	0.150
Death	0 (0.0)	1 (1.9)	1.000

*GPA* granulomatosis with polyangiitis, *HCP* hypertrophic cranial pachymeningitis, *GCs* glucocorticoids (including high, medium, and low doses), *MP* methylprednisolone, *CYC* cyclophosphamide (including all doses), *RTX* rituximab, *IVIG* intravenous immunoglobulin

^*^p < 0.05

## Discussion

HCP is considered an uncommon but important feature that occurs most frequently among GPA patients with hearing loss and visual impairment, with a lower frequency of pulmonary and renal involvement [2]. Because HCP is the most significant indicator of CNS involvement in GPA, it is usually associated with higher disease activity. Therefore, more intensive treatment is required.

HCP is mainly characterized by headache and cranial nerve palsy. Likewise, in this study, we found that headache, which is related to inflammation in pain-sensitive areas and sometimes intracranial hypertension, was the most frequent presenting symptom. Although headache is not a specific manifestation, unexplained headache, especially when combined with focal or generalized neurological deficits, sinusitis, otitis media, or mastoiditis, generally indicates GPA-related HCP. The second most common neurological manifestation was cranial nerve palsy. Three of the nerves most commonly involved were optic, facial, and vestibulocochlear nerves. Brain nerve impairment is related to the location of dural thickening. Cerebral vasculitis occurs in approximately 3 to 5% of patients with GPA [9], resulting in ischemic or hemorrhagic impairment, such as transient ischemic attack or stroke, organic confusion, seizure, and spontaneous subdural hematomas (SDHs). Only one case of intracranial sinus thrombosis, seizure, and stroke has been reported, and none of the patients with GPA-related HCP had SDHs, whereas a previous study showed a higher proportion of patients with SDHs [10]. Pituitary dysfunction is an uncommon CNS complication of GPA, and a previous study showed that only 12 (3.9%) of 304 patients were diagnosed with GPA during PUMCH administration [11]. Pituitary involvement is relatively easy to recognize compared to HCP due to polydipsia, polyuria, and hormonal dysfunction. Typically, more time is needed to diagnose patients with GPA-related HCP because the neurological features present at the onset of the disease usually challenge clinicians at the first diagnosis. All of these features are significant for neurologists to identify GPA-related HCP early when various CNS disorders and systemic manifestations are not well understood. Other general causes of HCP are idiopathic hypertrophic pachymeningitis, infection, trauma, malignancy, and other autoimmune diseases, such as IgG4-RD [12].

Contrast-enhanced MRI is the primary diagnostic technique used to detect dural thickening, as a dura mater biopsy is difficult to obtain. T1- or T2-weighted imaging can detect localized or widespread dural thickening with low or equal signal on T1- or T2-weighted imaging, as well as fattening and meningeal enhancement on the T1 gadolinium enhancement sequence. For patients with GPA, the intracranial dura mater is more often infiltrated than the spinal dura mater [9]. In patients with GPA-related HCP, diffuse linear thickening of the convexity is most common. The results of this study also revealed that convexity was the most common area involved, followed by tentorium, skull base, falx, and sellar region. Pathological examination of dural tissue usually reveals fibrosis, granulomatous vasculitis, and granulomatous inflammation with the presence of polykaryocyte Langhans giant cells [13].

Our study confirmed that conductive or sensorineural hearing loss and decreased vision or sudden visual loss were independently associated with GPA-related HCP. Notably, most patients had mixed conductive-sensorineural hearing loss, underscoring the importance of recognizing hearing loss as a noteworthy clinical symptom regardless of its classification. We reported some similar results to previous studies. Visual involvement was observed with a higher frequency in GPA patients with HCP compared to those without [2]. Sudden visual loss may be associated with optic nerve impairment. HCP was more prevalent in patients with otitis media with ANCA-associated vasculitis (OMAAV) than in the non-OMAAV group [14, 15]. Patients with exudative otitis media and mastoiditis had a higher incidence of meningeal involvement, along with indications of temporal or occipital enhancement on MRIs and signs of necrotizing granulomatosis [3, 16]. Granulation or effusion in the middle ear spreading to the dura mater is thought to be a probable cause of HCP [15]. Additionally, Shimojima et al. demonstrated that sudden visual loss and conductive hearing loss were significantly recognized in patients with AAV-associated HP, suggesting that inflammatory lesions proximal to CNS may play a pivotal role in the pathogenesis of AAV-associated HP [8]. Conversely, patients with GPA-related HCP were less likely to have pulmonary and renal involvement [2], which can potentially lead to misdiagnosis as localized GPA without identifying life-threatening organ dysfunction at the first diagnosis. All of these features provide new insights into the diagnosis and indicate the need for further intensive examination of HCP, including enhanced MRI and additional biopsies.

PR3-ANCA was predominantly found in patients with GPA without HCP. However, the gap between PR3-ANCA and MPO-ANCA positivity rates narrowed in patients with HCP. MPO-ANCA positivity was frequently detected in patients diagnosed with GPA and HCP, consistent with a previous study [17]. Another study showed that HP patients had significantly higher PR3-ANCA positivity compared to those without HP [15], which aligns with our research because GPA was more frequently observed in patients with HP [8], and PR3-ANCA positivity is a crucial criterion for GPA. The specificity of ANCA for either PR3 or MPO has recently been proposed for disease classification [18], as serologic specificity is related to both clinical characteristics and prognosis. Notably, there was a higher proportion of MPO-ANCA in the HCP group compared to the non-HCP group. The relatively increased frequency of MPO-ANCA detection in GPA-HCP warrants further investigation. Approximately 4% of patients with a clinical diagnosis of GPA-related HCP have tested negative for ANCA [19]. However, the proportion of patients with negative ANCA results in this study was slightly higher at 15.4%.

Because CNS vasculitis is defined a severe and active disease [20], more aggressive therapeutics are preferred. Glucocorticoids combined with CYC are the mainstay treatment for GPA. All patients with GPA-related HCP were treated with induction therapy as previously mentioned. Patients in the GPA-related HCP group received more intense methylprednisolone pulse treatment than those in the control group. High-dose IVIG therapy and intrathecal injections of dexamethasone and methotrexate were frequently used to treat patients with CNS involvement, although the efficacy of these treatments is still uncertain. RTX, a monoclonal anti-CD20 antibody, has been shown to induce clinical and imaging remission in patients with GPA-related HCP who have failed high-dose glucocorticoids combined with immunosuppressants [21]. RTX has been recommended as induction therapy at the first onset of GPA [7]. In patients with GPA-related HCP, RTX may be effective during both the induction and relapse phases, as three patients who had not previously achieved remission and two who had relapsed benefited from RTX in our study. However, RTX is not as commonly used as CTX due to a lack of data for patients with GPA and HCP. Biological agents such as RTX require long-term follow-up. Since most patients admitted to our hospital had more severe conditions, the relapse rate for GPA patients with HCP was reported to be higher in our center than reported in the literature [3]. Additionally, the relapse rate in the HCP group was somewhat higher than in the non-HCP group, although there was no statistically significant difference between the two groups. HCP is suspected of being prone to relapse due to recurrent ENT involvement, and larger clinical trials are necessary to support this assumption.

Our study had some limitations that should be acknowledged. First, as our study was a single-center study, the enrollment of patients with GPA-related HCP is limited, and our sample size may not be sufficient to identify all potential risk factors. Patients in our center usually tend to have more severe conditions and are more difficult to diagnose than those in other centers; therefore, our results may not completely represent the larger population with the disease. Second, the observed differences in baseline characteristics suggest that our matching strategy may have impacted the study outcomes. Lastly, the long-term follow-up data was unavailable. A large-scale cohort study is needed to explore these conclusive prognoses.

## Conclusions

GPA-related HCP was a special form of GPA with less pulmonary and renal involvement, more incidence of hearing and visual loss, and severe neurological involvement, mainly including headaches, cranial nerve palsy, and pituitary involvement, which delayed the diagnosis. PR3-ANCA negativity, conductive or sensorineural hearing loss, and decreased vision or sudden visual loss were independently associated with GPA-related HCP. These clinical features may serve as predictive indicators for diagnosing GPA-related HCP.

### Supplementary Information

**Additional file 1: Table S1.** Supplementary data on binary logistic regression analysis for GPA patients with HCP (method ‘forward conditional’). **Table S2.** Binary logistic regression analysis for GPA patients with HCP (method ‘enter’).

## Data Availability

The data is available in this article, and further inquiries can be directed to the corresponding authors.

## Abbreviations

GPA: Granulomatosis with polyangiitis
HCP: Hypertrophic cranial pachymeningitis
MPO-ANCA: Myeloperoxidase–antineutrophil cytoplasmic antibody
PR3-ANCA: Proteinase 3–antineutrophil cytoplasmic antibody
ANCA: Antineutrophil cytoplasmic antibody
BVAS: Birmingham Vasculitis Activity Score
FFS: Five-factor score
MRI: Magnetic resonance image
CNS: Central nervous system
PUMCH: Peking Union Medical College Hospital
ACR: American College of Rheumatology
EULAR: European League Against Rheumatism
CSF: Cerebrospinal fluid
ESR: Erythrocyte sedimentation rate
hsCRP: Hypersensitive C-reactive protein
WBC: White blood cells
ENT: Ear, nose, and throat
SDH: Spontaneous subdural hematoma
IVIG: Intravenous immunoglobulin
RTX: Rituximab

## References

[1] De Luna G, Terrier B, Kaminsky P, Le Quellec A, Maurier F, Solans R (2015). Central nervous system involvement of granulomatosis with polyangiitis: clinical-radiological presentation distinguishes different outcomes. Rheumatology (Oxford).

[2] Choi HA, Lee MJ, Chung CS (2017). Characteristics of hypertrophic pachymeningitis in patients with granulomatosis with polyangiitis. J Neurol.

[3] Sakairi T, Sakurai N, Nakasatomi M, Ikeuchi H, Kaneko Y, Maeshima A (2019). Hypertrophic pachymeningitis associated with antineutrophil cytoplasmic antibody-associated vasculitis: a case series of 15 patients. Scand J Rheumatol.

[4] Leavitt RY, Fauci AS, Bloch DA, Michel BA, Hunder GG, Arend WP (1990). The American College of Rheumatology 1990 criteria for the classification of Wegener’s granulomatosis. Arthritis Rheum.

[5] Watts R, Lane S, Hanslik T, Hauser T, Hellmich B, Koldingsnes W (2007). Development and validation of a consensus methodology for the classification of the ANCA-associated vasculitides and polyarteritis nodosa for epidemiological studies. Ann Rheum Dis.

[6] Grayson PA-O, Ponte CA-O, Suppiah R, Robson JA-O, Craven A, Judge AA-O (2022). 2022 American College of Rheumatology/European Alliance of Associations for rheumatology classification criteria for eosinophilic granulomatosis with polyangiitis. Ann Rheum Dis..

[7] Hellmich B, Sanchez-Alamo B, Schirmer JH, Berti A, Blockmans D, Cid MC, et al. EULAR recommendations for the management of ANCA-associated vasculitis: 2022 update. Ann Rheum Dis. 2023. 10.1136/ard-2022-223764 36927642

[8] Shimojima Y, Kishida D, Ichikawa T, Kida T, Yajima N, Omura S, Nakagomi D, Abe Y, Kadoya M, Takizawa N (2022). Hypertrophic pachymeningitis in ANCA-associated vasculitis: a cross-sectional and multi-institutional study in Japan (J-CANVAS). Arthritis Res Ther.

[9] Holle JU, Gross WL (2011). Neurological involvement in Wegener’s granulomatosis. Curr Opin Rheumatol.

[10] Shimojima Y, Kishida D, Hineno A, Yazaki M, Sekijima Y, Ikeda S-I (2017). Hypertrophic pachymeningitis is a characteristic manifestation of granulomatosis with polyangiitis: a retrospective study of anti-neutrophil cytoplasmic antibody-associated vasculitis. Int J Rheum Dis.

[11] Gu Y, Sun X, Peng M, Zhang T, Shi J, Mao J (2019). Pituitary involvement in patients with granulomatosis with polyangiitis: case series and literature review. Rheumatol Int.

[12] Masson C, Boukriche Y, Colombani JM (2021). Inflammatory hypertrophic cranial pachymeningitis. Presse Med.

[13] Arnaoutoglou MA, Xerras CG, Kalevrosoglou IK, Rafailidis VD, Notas KP, Tegos TI (2018). Headache linked to intracranial hypertension and hypertrophic pachymeningitis as the initial and dominant presentation of granulomatosis with polyangiitis. Case report and review of the recent literature. Headache..

[14] Hosokawa Y, Okada M, Suemori K, Hamaguchi N, Miyoshi KI, Takagi T (2021). The association between ear involvement and clinical features and prognosis in ANCA-associated vasculitis. Auris Nasus Larynx.

[15] Harabuchi Y, Kishibe K, Tateyama K, Morita Y, Yoshida N, Kunimoto Y (2017). Clinical features and treatment outcomes of otitis media with antineutrophil cytoplasmic antibody (ANCA)-associated vasculitis (OMAAV): a retrospective analysis of 235 patients from a nationwide survey in Japan. Mod Rheumatol.

[16] Di Comite G, Bozzolo EP, Praderio L, Tresoldi M, Sabbadini MG (2006). Meningeal involvement in Wegener’s granulomatosis is associated with localized disease. Clin Exp Rheumatol.

[17] Yokoseki A, Saji E, Arakawa M, Kosaka T, Hokari M, Toyoshima Y (2014). Hypertrophic pachymeningitis: significance of myeloperoxidase anti-neutrophil cytoplasmic antibody. Brain.

[18] Wallace ZS, Stone JH (2019). Personalized medicine in ANCA-associated vasculitis ANCA specificity as the guide?. Front Immunol.

[19] Miloslavsky EM, Lu N, Unizony S, Choi HK, Merkel PA, Seo P (2016). Myeloperoxidase-antineutrophil cytoplasmic antibody (ANCA)-positive and ANCA-negative patients with granulomatosis with polyangiitis (Wegener’s): distinct patient subsets. Arthritis Rheumatol.

[20] Chung SA, Langford CA, Maz M, Abril A, Gorelik M, Guyatt G (2021). 2021 American College of Rheumatology/Vasculitis Foundation Guideline for the management of antineutrophil cytoplasmic antibody-associated vasculitis. Arthritis Rheumatol.

[21] Just SA, Knudsen JB, Nielsen MK, Junker P (2011). Wegener’s granulomatosis presenting with pachymeningitis: clinical and imaging remission by rituximab. ISRN Rheumatol..

