# Supplementary material for: Clinical characteristics of hypertrophic cranial pachymeningitis in granulomatosis with polyangiitis: a retrospective single-center study in China
Source: Arthritis Res Ther. 2024 Jan 2;26:6. doi: 10.1186/s13075-023-03239-9 (PMC10759605; doi:10.1186/s13075-023-03239-9)
Supplement: Supplementary file 1 — Additional file 1: Table S1. Supplementary data on binary logistic regression analysis for GPA patients with HCP (method ‘forward conditional’). Table S2. Binary logistic regression analysis for GPA patients with HCP (method ‘enter’). [file 13075_2023_3239_MOESM1_ESM.docx]

**Supplementary Tables**

**Tables S1** Supplementary data on binary logistic regression analysis for GPA patients with HCP (method ‘forward conditional’)

| **Variables not in the equation** | P value |
| --- | --- |
| Conjunctivitis/ uveitis/ blepharitis/ keratitis | 0.101 |
| Mastoiditis | 0.127 |
| Pulmonary involvement | 0.182 |
| Renal involvement | 0.542 |

**Tables S2** Binary logistic regression analysis for GPA patients with HCP (method ‘enter’)

| **All the variables in the equation** | **Beta** | **S.E** | **P value** | **OR (95%CI)** |  |
| --- | --- | --- | --- | --- | --- |
| PR3-ANCA negativity | 2.172 | 0.804 | 0.007* | 8.774 (1.816 – 42.387) |  |
| Conductive/sensorineural hearing loss | 1.976 | 0.949 | 0.037* | 7.215 (1.123 - 46.370) |  |
| Decreased vision/sudden visual loss | 2.121 | 0.963 | 0.028* | 8.343 (1.264 - 55.056) |  |
| Conjunctivitis/ uveitis/ blepharitis/ keratitis | -1.766 | 1.236 | 0.153 | 0.171 (0.015 – 1.929) |  |
| Mastoiditis | 0.931 | 0.726 | 0.200 | 2.537 (0.611 - 10.526) |  |
| Pulmonary involvement | -0.722 | 0.794 | 0.363 | 0.486 (0.102 - 2.304) |  |
| Renal involvement | -0.353 | 0.943 | 0.708 | 0.703 (0.111 - 4.461) |  |

**p*＜0.05
